# Crystal structure of 4-(benzo[*d*]thia­zol-2-yl)-1,2-dimethyl-1*H*-pyrazol-3(2*H*)-one

**DOI:** 10.1107/S2056989024001257

**Published:** 2024-02-16

**Authors:** Heba A. Elboshi, Rasha A. Azzam, Galal H. Elgemeie, Peter G. Jones

**Affiliations:** a Chemistry Department, Faculty of Science, Helwan University, Cairo, Egypt; b Institut für Anorganische und Analytische Chemie, Technische Universität Braunschweig, Hagenring 30, D-38106 Braunschweig, Germany; Universität Greifswald, Germany

**Keywords:** crystal structure, benzo­thia­zole, pyrazolone, weak hydrogen bonds

## Abstract

In the title compound, C_12_H_11_N_3_OS, the inter­planar angle between the pyrazole and benzo­thia­zole rings is 3.31 (7)°. In the three-dimensional mol­ecular packing, the carbonyl oxygen acts as acceptor to four C—H donors (with one H⋯O as short as 2.25 Å), while one methyl hydrogen is part of the three-centre system H⋯(S, O). A double layer structure parallel to (  01) can be recognized as a subsection of the packing.

## Abstract 2

The ring systems of 4-(benzo[*d*]thia­zol-2-yl)-1,2-dimethyl-1*H*-pyrazol-3(2*H*)-one are almost coplanar. In the three-dimensional packing, the carbonyl oxygen accepts four weak hydrogen bonds.

## 1. Chemical context

Many natural heterocyclic compounds and pharmaceuticals involve benzo­thia­zole moieties and derivatives thereof, which are among the most significant heterocyclic compounds utilized in medicinal chemistry (Bonde *et al.*, 2015 ▸). In the search for novel and significant therapeutic drugs, benzo­thia­zoles have a wide range of established pharmacological properties (Wang *et al.*, 2009 ▸), and their derivatives include several structural variants (Rana *et al.*, 2008 ▸). The application of benzo­thia­zole derivatives in current research and related discoveries is a well-appreciated and quickly growing area of medicinal chemistry (Abdallah *et al.*, 2023 ▸). As an example, several drugs based on benzo­thia­zole derivatives have been widely utilized in clinical practice to treat a variety of disorders, with a marked therapeutic efficacy (Huang *et al.*, 2009 ▸).

In the course of our studies, intended to develop syntheses of benzo­thia­zole-based heterocycles for use as pharmaceuticals and pigments (Ahmed *et al.*, 2022 ▸, 2023 ▸), a variety of 2-pyrimidyl-, 2-pyridyl- and 2-thienyl-benzo­thia­zole compounds with encouraging cytotoxic action have recently been synthesized and their biological activity reported (Azzam *et al.*, 2017 ▸, 2019 ▸, 2022 ▸). 
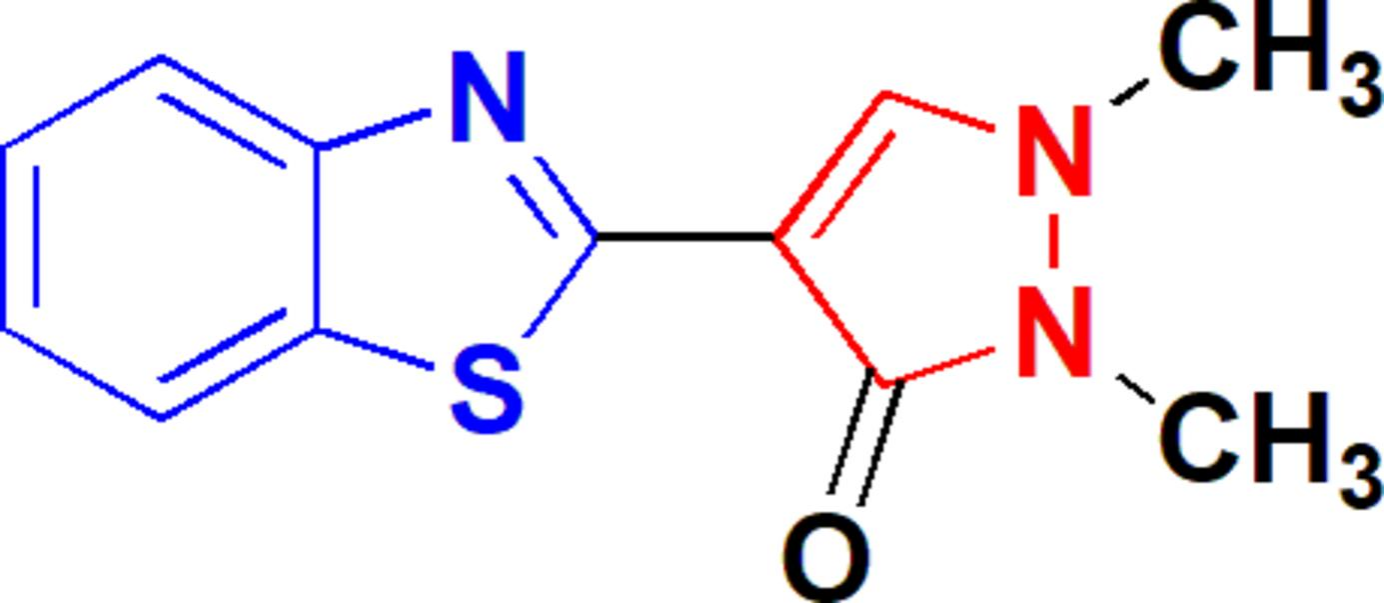


**Figure 1:**
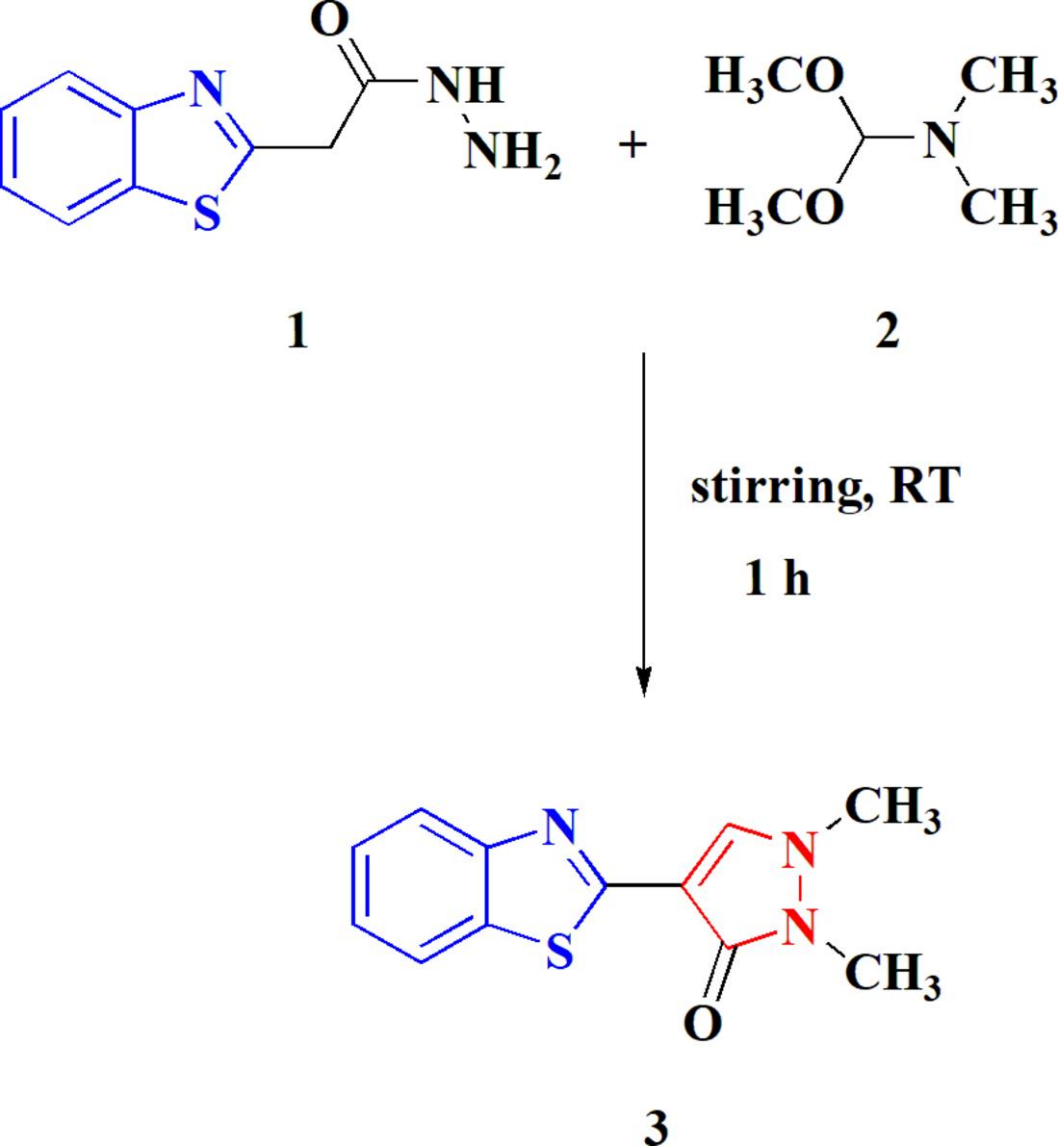
The synthesis of the title compound **3**.

In line with these findings and our prior research (Metwally *et al.*, 2022*a* ▸,*b* ▸), the aim of the current investigation was to design and create benzo­thia­zolyl-pyrazole hybrids. The reaction of 2-benzo­thia­zolyl acetohydrazide **1** with *N*,*N*-di­methyl­formamide dimethyl acetal **2** at room temperature led to the synthesis of the unexpected benzo­thia­zole-2-pyrazole derivative **3** in good yield (Fig. 1 ▸). The mechanism for the formation of **3** is currently under investigation. In order to establish the structure of the product unambiguously, its crystal structure was determined and is reported here.

## 2. Structural commentary

**Figure 2:**
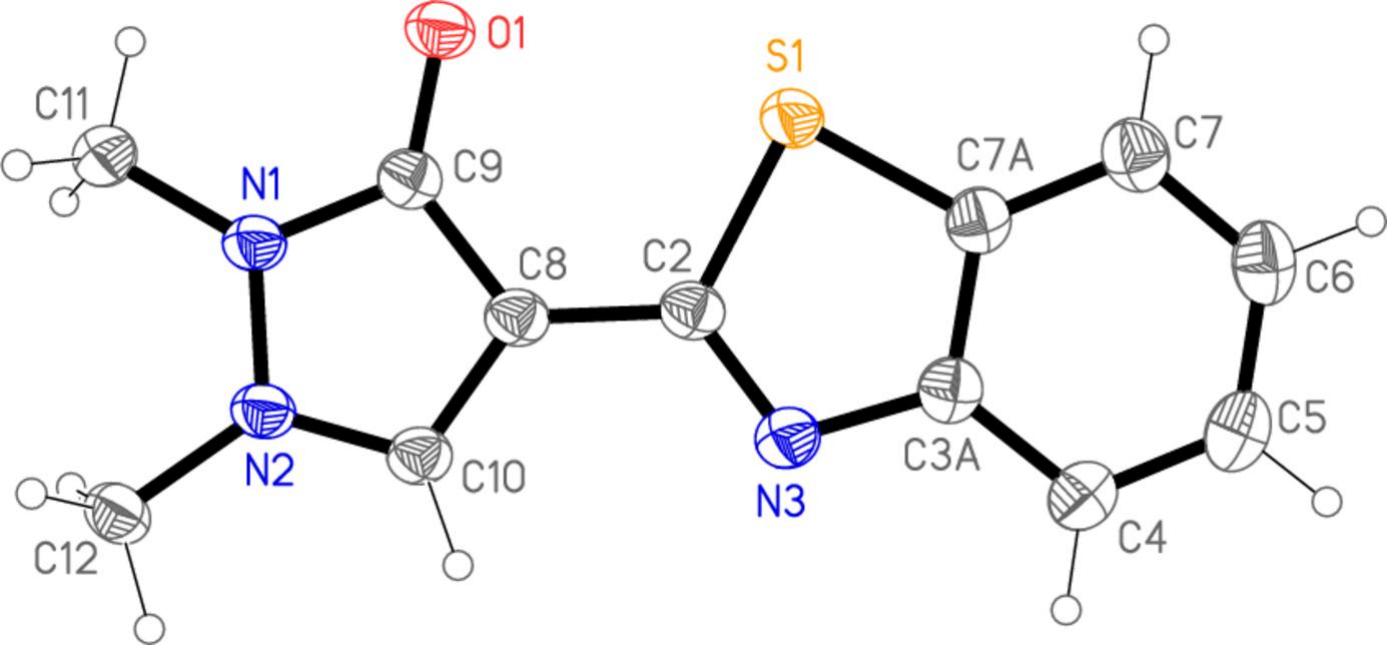
The structure of compound **3** in the crystal. Ellipsoids correspond to 50% probability levels.

The structure of compound **3** is shown in Fig. 2 ▸, with selected mol­ecular dimensions in Table 1 ▸. These may be regarded as normal, within the constraints of linked five-membered rings that necessarily lead to narrow angles within the rings and wide exocyclic angles [up to 127.48 (11)° for C10—C8—C2]. The mol­ecule is essentially planar (except for the methyl hydrogens); the least-squares plane through all non-H atoms has an r.m.s.d. of only 0.037 Å. If the ring systems are regarded separately, the pyrazole and benzo­thia­zole rings have r.m.s.d. values of 0.006 and 0.017 Å, respectively, and an inter­planar angle of 3.31 (7)°. The coplanarity leads to a short intra­molecular contact S1⋯O1 = 2.9797 (10) Å.

## 3. Supra­molecular features

**Figure 3:**
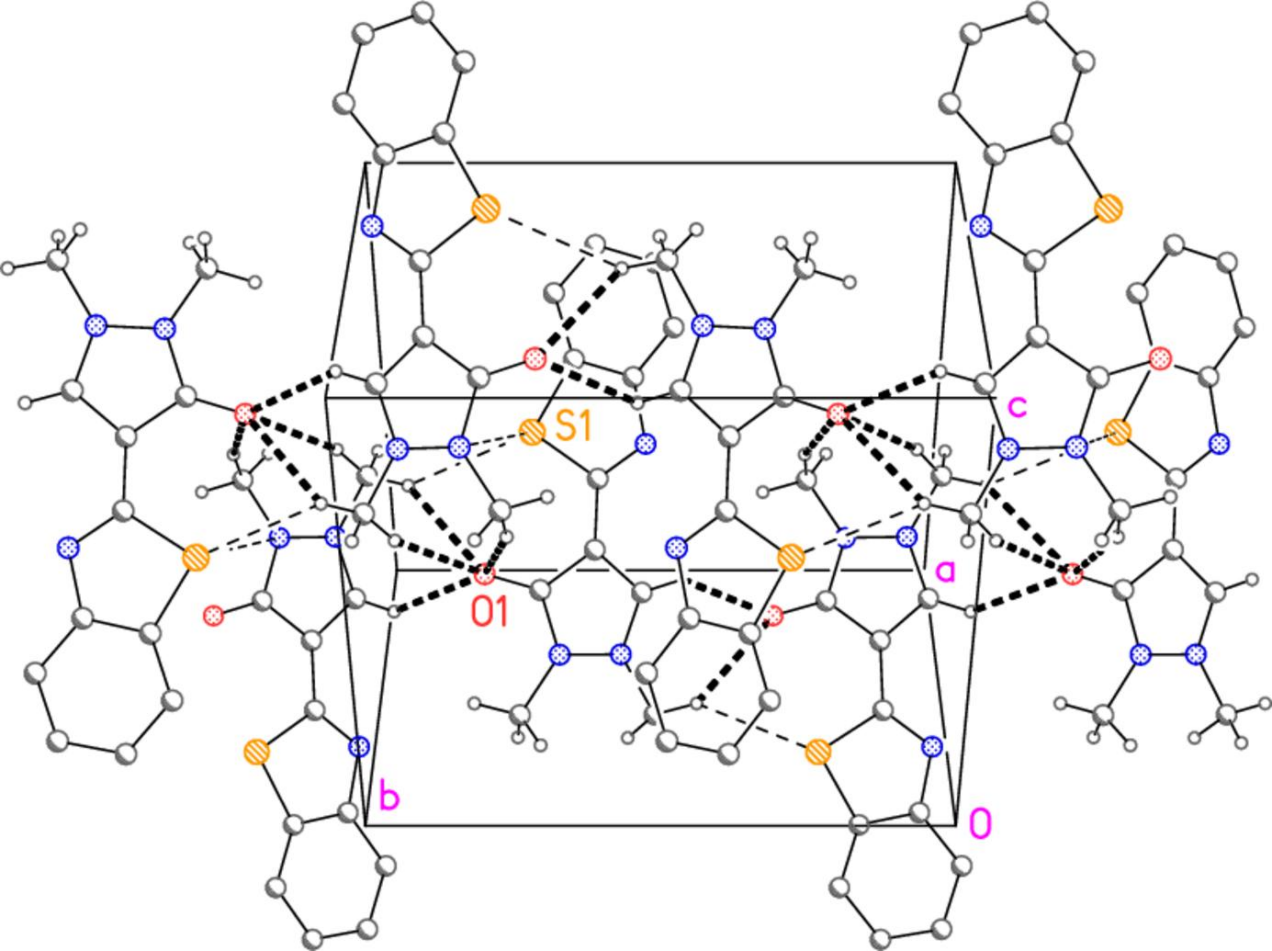
A section of the three-dimensional packing of compound **3**: two anti­parallel ribbons viewed perpendicular to ( 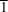 01). Contacts to O1 are shown as thick dashed lines and those to S1 as thin dashed lines. Hydrogen atoms not involved in hydrogen bonding are omitted. The atom labels indicate the asymmetric unit.

The mol­ecular packing involves five short contacts, four C—H⋯O1 and one C—H⋯S1, that are acceptably linear and may be regarded as ‘weak’ hydrogen bonds (Table 2 ▸). The donor atom H12*B* is part of a three-centre system with acceptors O1 and S1. The contact H12*C*⋯O1 is remarkably short at 2.25 Å. Additionally, there is a short contact S1⋯N1(*x*, 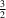 − *y*, 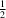 + *z*) = 3.4078 (11) Å. A section of the packing is shown in Fig. 3 ▸; a ribbon parallel to the *b* axis and its anti­parallel counterpart are shown, which form a double layer parallel to ( 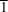 01). However, the mol­ecules are further linked parallel to the view direction to give a three-dimensional pattern. There are no *Cent*–*Cent* contacts shorter than 3.75 Å and no H⋯*Cent* contacts shorter than 2.99 Å (*Cent* = ring centroids).

## 4. Database survey

The search employed the routine ConQuest (Bruno *et al.*, 2002 ▸), part of Version 2023.3.0 of the Cambridge Structural Database (Groom *et al.*, 2016 ▸).

A search for other structures containing a linked pyrazolone/benzo­thia­zole unit as in **3** led to three hits: AZUPIV, with 1-Me, 2-Ph and 5-Me substituents on the pyrazolone ring (Chakib *et al.*, 2011 ▸), VABFIP (1-allyl, 2-Ph, 5-Me; Chakib *et al.*, 2010*a* ▸) and VABFOV (1-propynyl, 2-Ph, 5-Me; Chakib *et al.*, 2010*b* ▸). The inter­planar angles in these compounds are 6.1 (1), 7.9 (2) and 4.7 (1)°, respectively.

## 5. Synthesis and crystallization

A mixture of 2-benzo­thia­zolyl acetohydrazide **1** (0.01 mol) and *N*,*N*-di­methyl­formamide dimethyl acetal **2** (0.02 mol) was stirred at room temperature for 1 h. The excess acetal was distilled off under reduced pressure; the solid product was washed with a mixture of petroleum ether and diethyl ether (1:1) and then crystallized from ethanol.

Yellow solid; yield 85%; m.p. 414 K; IR (KBr, cm^−1^): ν 3068 (aromatic CH), 2930 (methyl CH), 1620 (C=O), 1598 (C=N); ^1^H NMR (400 MHz, DMSO-*d*_6_): δ 3.80 (*s*, 3H, CH_3_), 4.01 (*s*, 3H, CH_3_), 7.34 (*t*, *J* = 7.2 Hz, 1H, benzo­thia­zole H), 7.46 (*t*, *J* = 7.2 Hz, 1H, benzo­thia­zole H), 7.89 (*d*, *J* = 7.6 Hz, 1H, benzo­thia­zole H), 8.03 (*d*, *J* = 8.0 Hz, 1H, benzo­thia­zole H), 8.35 (*s*, 1H, pyrazolone H). Analysis calculated for C_12_H_11_N_3_OS (245.30): C 58.76, H 4.52, N 17.13, S 13.07. Found C 58.66, H 4.40, N 17.08, S 13.14%.

## 6. Refinement

Crystal data, data collection and structure refinement details are summarized in Table 3 ▸. The methyl groups were included as idealized rigid groups (C—H 0.98 Å, H—C—H 109.5°) allowed to rotate but not tip (command ‘AFIX 137’). Other hydrogen atoms were included using a riding model starting from calculated positions (C—H = 0.95 Å). The *U*_iso_(H) values were fixed at 1.5 × *U*_eq_ of the parent carbon atoms for the methyl group and 1.2 × *U*_eq_ for other hydrogens. One reflection clearly in error (*F*_o_^2^ >> *F*_c_^2^) was omitted from the refinement.

## Supplementary Material

Crystal structure: contains datablock(s) I, global. DOI: 10.1107/S2056989024001257/yz2050sup1.cif

Structure factors: contains datablock(s) I. DOI: 10.1107/S2056989024001257/yz2050Isup2.hkl

Supporting information file. DOI: 10.1107/S2056989024001257/yz2050Isup3.cml

CCDC reference: 2331586

Additional supporting information:  crystallographic information; 3D view; checkCIF report

## Figures and Tables

**Table 1:** Selected geometric parameters (Å, °)

S1—C7*A*	1.7374 (13)	N2—C10	1.3326 (16)
S1—C2	1.7673 (12)	N3—C2	1.3051 (16)
O1—C9	1.2468 (15)	N3—C3*A*	1.3879 (16)
N1—N2	1.3766 (14)	C2—C8	1.4348 (17)
N1—C9	1.3772 (16)	C3*A*—C7*A*	1.4058 (18)
			
C7*A*—S1—C2	88.85 (6)	C7—C7*A*—S1	128.77 (11)
N2—N1—C9	109.59 (10)	C3*A*—C7*A*—S1	109.37 (9)
C10—N2—N1	108.87 (10)	C10—C8—C2	127.48 (11)
C2—N3—C3*A*	110.45 (11)	C10—C8—C9	107.06 (11)
N3—C2—C8	124.55 (11)	C2—C8—C9	125.46 (11)
N3—C2—S1	115.65 (9)	O1—C9—N1	123.88 (11)
C8—C2—S1	119.80 (9)	O1—C9—C8	130.92 (12)
N3—C3*A*—C4	125.04 (12)	N1—C9—C8	105.19 (10)
N3—C3*A*—C7*A*	115.66 (11)	N2—C10—C8	109.28 (11)

**Table 2:** Hydrogen-bond geometry (Å, °)

*D*—H⋯*A*	*D*—H	H⋯*A*	*D*⋯*A*	*D*—H⋯*A*
C10—H10⋯O1^i^	0.95	2.38	3.2055 (15)	145
C11—H11*A*⋯O1^ii^	0.98	2.51	3.4368 (16)	158
C12—H12*B*⋯S1^i^	0.98	2.86	3.7142 (13)	146
C12—H12*B*⋯O1^i^	0.98	2.60	3.4696 (16)	148
C12—H12*C*⋯O1^ii^	0.98	2.25	3.1966 (16)	163

Symmetry codes: (i) 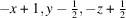 ; (ii) 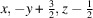 .

**Table 3:** Experimental details

Crystal data	
Chemical formula	C_12_H_11_N_3_OS
*M*_r_	245.30
Crystal system, space group	Monoclinic, *P*2_1_/*c*
Temperature (K)	100
*a*, *b*, *c* (Å)	8.78308 (12), 11.66215 (16), 11.00169 (15)
β (°)	97.9460 (12)
*V* (Å^3^)	1116.08 (3)
*Z*	4
Radiation type	Cu *K*α
μ (mm^−1^)	2.47
Crystal size (mm)	0.15 × 0.10 × 0.03

Data collection	
Diffractometer	XtaLAB Synergy
Absorption correction	Multi-scan (*CrysAlis PRO*; Rigaku OD, 2021 ▸)
*T*_min_, *T*_max_	0.731, 1.000
No. of measured, independent and observed [*I* > 2σ(*I*)] reflections	46018, 2367, 2280
*R*_int_	0.037
(sin θ/λ)_max_ (Å^−1^)	0.634

Refinement	
*R*[*F* ^2^ > 2σ(*F* ^2^)], *wR*(*F* ^2^), *S*	0.030, 0.081, 1.07
No. of reflections	2367
No. of parameters	156
H-atom treatment	H-atom parameters constrained
Δρ_max_, Δρ_min_ (e Å^−3^)	0.28, −0.36

Computer programs: *CrysAlis PRO* (Rigaku OD, 2021 ▸), *SHELXT* (Sheldrick, 2015*a* ▸), *SHELXL2019/3* (Sheldrick, 2015*b* ▸) and *XP* (Bruker, 1998 ▸).

## supplementary crystallographic information

### Crystal data

**Table:**

C_12_H_11_N_3_OS	*F*(000) = 512
*M**_r_* = 245.30	*D*_x_ = 1.460 Mg m^−^^3^
Monoclinic, *P*2_1_/*c*	Cu *K*α radiation, λ = 1.54184 Å
*a* = 8.78308 (12) Å	Cell parameters from 32695 reflections
*b* = 11.66215 (16) Å	θ = 5.1–77.4°
*c* = 11.00169 (15) Å	µ = 2.47 mm^−^^1^
β = 97.9460 (12)°	*T* = 100 K
*V* = 1116.08 (3) Å^3^	Plate, pale yellow
*Z* = 4	0.15 × 0.10 × 0.03 mm

### Data collection

**Table:**

XtaLAB Synergy diffractometer	2367 independent reflections
Radiation source: micro-focus sealed X-ray tube, PhotonJet (Cu) X-ray Source	2280 reflections with *I* > 2σ(*I*)
Mirror monochromator	*R*_int_ = 0.037
Detector resolution: 10.0000 pixels mm^-1^	θ_max_ = 77.6°, θ_min_ = 5.1°
ω scans	*h* = −11→11
Absorption correction: multi-scan (CrysAlisPro; Rigaku OD, 2021)	*k* = −14→14
*T*_min_ = 0.731, *T*_max_ = 1.000	*l* = −13→13
46018 measured reflections	

### Refinement

**Table:**

Refinement on *F*^2^	Primary atom site location: dual
Least-squares matrix: full	Hydrogen site location: inferred from neighbouring sites
*R*[*F*^2^ > 2σ(*F*^2^)] = 0.030	H-atom parameters constrained
*wR*(*F*^2^) = 0.081	*w* = 1/[σ^2^(*F*_o_^2^) + (0.0434*P*)^2^ + 0.4017*P*] where *P* = (*F*_o_^2^ + 2*F*_c_^2^)/3
*S* = 1.07	(Δ/σ)_max_ = 0.001
2367 reflections	Δρ_max_ = 0.28 e Å^−^^3^
156 parameters	Δρ_min_ = −0.36 e Å^−^^3^
0 restraints	

### Special details

**Table:**

Geometry. All esds (except the esd in the dihedral angle between two l.s. planes) are estimated using the full covariance matrix. The cell esds are taken into account individually in the estimation of esds in distances, angles and torsion angles; correlations between esds in cell parameters are only used when they are defined by crystal symmetry. An approximate (isotropic) treatment of cell esds is used for estimating esds involving l.s. planes.

### Fractional atomic coordinates and isotropic or equivalent isotropic displacement parameters (Å^2^)

**Table:**

	*x*	*y*	*z*	*U*_iso_*/*U*_eq_	
S1	0.63680 (4)	0.71933 (2)	0.56971 (3)	0.02849 (11)	
O1	0.40109 (11)	0.79924 (7)	0.36636 (8)	0.0311 (2)	
N1	0.33946 (12)	0.67380 (9)	0.20375 (9)	0.0266 (2)	
N2	0.39212 (12)	0.56914 (8)	0.16939 (9)	0.0268 (2)	
N3	0.73240 (12)	0.52608 (9)	0.48701 (10)	0.0288 (2)	
C2	0.63324 (14)	0.60863 (10)	0.45966 (11)	0.0259 (2)	
C3A	0.82145 (14)	0.54730 (11)	0.59907 (12)	0.0286 (3)	
C4	0.94097 (16)	0.47710 (12)	0.65366 (13)	0.0350 (3)	
H4	0.964387	0.407342	0.615676	0.042*	
C5	1.02447 (16)	0.51076 (13)	0.76368 (13)	0.0382 (3)	
H5	1.106443	0.463964	0.800905	0.046*	
C6	0.98995 (16)	0.61274 (13)	0.82092 (13)	0.0378 (3)	
H6	1.049479	0.634461	0.896126	0.045*	
C7	0.87033 (16)	0.68266 (12)	0.76977 (12)	0.0339 (3)	
H7	0.845688	0.751223	0.809420	0.041*	
C7A	0.78751 (14)	0.64905 (11)	0.65836 (11)	0.0284 (3)	
C8	0.52570 (14)	0.61387 (10)	0.34907 (11)	0.0258 (2)	
C9	0.42066 (14)	0.70641 (10)	0.31446 (11)	0.0258 (2)	
C10	0.50225 (14)	0.53273 (10)	0.25637 (11)	0.0267 (3)	
H10	0.556198	0.462239	0.255040	0.032*	
C11	0.22779 (15)	0.74119 (11)	0.12468 (12)	0.0313 (3)	
H11A	0.260232	0.747813	0.043243	0.047*	
H11B	0.220412	0.817829	0.159925	0.047*	
H11C	0.127222	0.703488	0.117398	0.047*	
C12	0.32435 (15)	0.51086 (11)	0.05843 (12)	0.0301 (3)	
H12A	0.214516	0.498538	0.061218	0.045*	
H12B	0.375258	0.436717	0.052279	0.045*	
H12C	0.337515	0.557948	−0.013158	0.045*	

### Atomic displacement parameters (Å^2^)

**Table:**

	*U*^11^	*U*^22^	*U*^33^	*U*^12^	*U*^13^	*U*^23^
S1	0.03690 (19)	0.02172 (17)	0.02606 (17)	0.00157 (11)	0.00148 (12)	−0.00057 (10)
O1	0.0411 (5)	0.0228 (4)	0.0290 (4)	0.0033 (4)	0.0038 (4)	−0.0025 (3)
N1	0.0317 (5)	0.0205 (5)	0.0273 (5)	0.0015 (4)	0.0028 (4)	−0.0008 (4)
N2	0.0334 (5)	0.0198 (5)	0.0267 (5)	0.0006 (4)	0.0030 (4)	−0.0013 (4)
N3	0.0314 (5)	0.0253 (5)	0.0295 (5)	0.0014 (4)	0.0038 (4)	0.0007 (4)
C2	0.0308 (6)	0.0210 (5)	0.0265 (6)	−0.0019 (4)	0.0062 (5)	0.0007 (4)
C3A	0.0287 (6)	0.0278 (6)	0.0294 (6)	−0.0021 (5)	0.0044 (5)	0.0023 (5)
C4	0.0338 (6)	0.0336 (7)	0.0371 (7)	0.0045 (5)	0.0033 (5)	0.0025 (5)
C5	0.0315 (7)	0.0431 (8)	0.0386 (7)	0.0022 (6)	−0.0002 (5)	0.0073 (6)
C6	0.0355 (7)	0.0437 (8)	0.0321 (7)	−0.0076 (6)	−0.0025 (5)	0.0034 (6)
C7	0.0380 (7)	0.0318 (7)	0.0312 (6)	−0.0065 (5)	0.0025 (5)	0.0005 (5)
C7A	0.0309 (6)	0.0258 (6)	0.0286 (6)	−0.0034 (5)	0.0046 (5)	0.0037 (5)
C8	0.0316 (6)	0.0204 (5)	0.0258 (6)	−0.0008 (4)	0.0051 (5)	0.0010 (4)
C9	0.0305 (6)	0.0228 (6)	0.0247 (6)	−0.0013 (4)	0.0054 (5)	0.0015 (4)
C10	0.0324 (6)	0.0206 (6)	0.0273 (6)	0.0003 (5)	0.0045 (5)	0.0017 (5)
C11	0.0353 (7)	0.0270 (6)	0.0303 (6)	0.0049 (5)	0.0000 (5)	−0.0002 (5)
C12	0.0374 (7)	0.0239 (6)	0.0281 (6)	−0.0029 (5)	0.0014 (5)	−0.0023 (5)

### Geometric parameters (Å, º)

**Table:**

S1—C7A	1.7374 (13)	C5—C6	1.398 (2)
S1—C2	1.7673 (12)	C5—H5	0.9500
O1—C9	1.2468 (15)	C6—C7	1.386 (2)
N1—N2	1.3766 (14)	C6—H6	0.9500
N1—C9	1.3772 (16)	C7—C7A	1.3923 (18)
N1—C11	1.4493 (16)	C7—H7	0.9500
N2—C10	1.3326 (16)	C8—C10	1.3856 (17)
N2—C12	1.4511 (15)	C8—C9	1.4365 (17)
N3—C2	1.3051 (16)	C10—H10	0.9500
N3—C3A	1.3879 (16)	C11—H11A	0.9800
C2—C8	1.4348 (17)	C11—H11B	0.9800
C3A—C4	1.3995 (18)	C11—H11C	0.9800
C3A—C7A	1.4058 (18)	C12—H12A	0.9800
C4—C5	1.383 (2)	C12—H12B	0.9800
C4—H4	0.9500	C12—H12C	0.9800
			
C7A—S1—C2	88.85 (6)	C7A—C7—H7	121.1
N2—N1—C9	109.59 (10)	C7—C7A—C3A	121.85 (12)
N2—N1—C11	122.76 (10)	C7—C7A—S1	128.77 (11)
C9—N1—C11	127.29 (10)	C3A—C7A—S1	109.37 (9)
C10—N2—N1	108.87 (10)	C10—C8—C2	127.48 (11)
C10—N2—C12	128.87 (10)	C10—C8—C9	107.06 (11)
N1—N2—C12	122.15 (10)	C2—C8—C9	125.46 (11)
C2—N3—C3A	110.45 (11)	O1—C9—N1	123.88 (11)
N3—C2—C8	124.55 (11)	O1—C9—C8	130.92 (12)
N3—C2—S1	115.65 (9)	N1—C9—C8	105.19 (10)
C8—C2—S1	119.80 (9)	N2—C10—C8	109.28 (11)
N3—C3A—C4	125.04 (12)	N2—C10—H10	125.4
N3—C3A—C7A	115.66 (11)	C8—C10—H10	125.4
C4—C3A—C7A	119.29 (12)	N1—C11—H11A	109.5
C5—C4—C3A	119.03 (13)	N1—C11—H11B	109.5
C5—C4—H4	120.5	H11A—C11—H11B	109.5
C3A—C4—H4	120.5	N1—C11—H11C	109.5
C4—C5—C6	120.93 (13)	H11A—C11—H11C	109.5
C4—C5—H5	119.5	H11B—C11—H11C	109.5
C6—C5—H5	119.5	N2—C12—H12A	109.5
C7—C6—C5	121.11 (13)	N2—C12—H12B	109.5
C7—C6—H6	119.4	H12A—C12—H12B	109.5
C5—C6—H6	119.4	N2—C12—H12C	109.5
C6—C7—C7A	117.78 (13)	H12A—C12—H12C	109.5
C6—C7—H7	121.1	H12B—C12—H12C	109.5
			
C9—N1—N2—C10	1.41 (13)	C4—C3A—C7A—S1	179.30 (10)
C11—N1—N2—C10	174.94 (11)	C2—S1—C7A—C7	177.82 (13)
C9—N1—N2—C12	177.72 (11)	C2—S1—C7A—C3A	−0.88 (9)
C11—N1—N2—C12	−8.75 (17)	N3—C2—C8—C10	3.3 (2)
C3A—N3—C2—C8	178.92 (11)	S1—C2—C8—C10	−176.73 (10)
C3A—N3—C2—S1	−1.06 (13)	N3—C2—C8—C9	−176.88 (11)
C7A—S1—C2—N3	1.17 (10)	S1—C2—C8—C9	3.10 (17)
C7A—S1—C2—C8	−178.81 (10)	N2—N1—C9—O1	177.75 (11)
C2—N3—C3A—C4	−178.36 (12)	C11—N1—C9—O1	4.6 (2)
C2—N3—C3A—C7A	0.34 (15)	N2—N1—C9—C8	−1.34 (13)
N3—C3A—C4—C5	177.54 (13)	C11—N1—C9—C8	−174.50 (11)
C7A—C3A—C4—C5	−1.12 (19)	C10—C8—C9—O1	−178.19 (13)
C3A—C4—C5—C6	0.6 (2)	C2—C8—C9—O1	1.9 (2)
C4—C5—C6—C7	0.5 (2)	C10—C8—C9—N1	0.80 (13)
C5—C6—C7—C7A	−1.2 (2)	C2—C8—C9—N1	−179.06 (11)
C6—C7—C7A—C3A	0.65 (19)	N1—N2—C10—C8	−0.86 (13)
C6—C7—C7A—S1	−177.91 (10)	C12—N2—C10—C8	−176.86 (12)
N3—C3A—C7A—C7	−178.29 (11)	C2—C8—C10—N2	179.88 (12)
C4—C3A—C7A—C7	0.49 (19)	C9—C8—C10—N2	0.03 (14)
N3—C3A—C7A—S1	0.52 (14)		

### Hydrogen-bond geometry (Å, º)

**Table:**

*D*—H···*A*	*D*—H	H···*A*	*D*···*A*	*D*—H···*A*
C10—H10···O1^i^	0.95	2.38	3.2055 (15)	145
C11—H11*A*···O1^ii^	0.98	2.51	3.4368 (16)	158
C12—H12*B*···S1^i^	0.98	2.86	3.7142 (13)	146
C12—H12*B*···O1^i^	0.98	2.60	3.4696 (16)	148
C12—H12*C*···O1^ii^	0.98	2.25	3.1966 (16)	163

Symmetry codes: (i) −*x*+1, *y*−1/2, −*z*+1/2; (ii) *x*, −*y*+3/2, *z*−1/2.
